# Retracted: Effect of Radical Laparoscopic Surgery and Conventional Open Surgery on Surgical Outcomes, Complications, and Prognosis in Elderly Patients with Bladder Cancer

**DOI:** 10.1155/2023/9835627

**Published:** 2023-01-11

**Authors:** Evidence-Based Complementary and Alternative Medicine

*Evidence-Based Complementary and Alternative Medicine* has retracted the article titled “Effect of Radical Laparoscopic Surgery and Conventional Open Surgery on Surgical Outcomes, Complications, and Prognosis in Elderly Patients with Bladder Cancer” [1] due to concerns that the peer review process has been compromised.

Following an investigation conducted by the Hindawi Research Integrity team [2], significant concerns were identified with the peer reviewers assigned to this article; the investigation has concluded that the peer review process was compromised. We therefore can no longer trust the peer review process, and the article is being retracted with the agreement of the Chief Editor.

The authors agree to the retraction.
